# Polymer Composite with Enhanced Thermal Conductivity and Insulation Properties through Aligned Al_2_O_3_ Fiber

**DOI:** 10.3390/polym14122374

**Published:** 2022-06-12

**Authors:** Sijiao Wang, Mengmeng Chen, Kaiming Cao

**Affiliations:** 1 Department of Mechatronic Engineering, School of Mechanical and Electrical Engineering, Xi’an Technological University, Xi’an 710021, China; mengchen0206@163.com (M.C.); kaiming1995@163.com (K.C.); 2 Department of Polymer Science and Engineering, School of Chemistry and Biological Engineering, University of Science and Technology Beijing, Beijing 100083, China; 3 State Key Laboratory of Power System, Department of Electrical Engineering, Tsinghua University, Beijing 100084, China

**Keywords:** Al_2_O_3_/LDPE composites, electrical properties, space charge, thermal conductivity

## Abstract

Thermoplastic polyolefins, such as polyethylene (PE), are traditionally one of the most widely used polymer classes with applications in the electric industry, and their nanocomposites have caught the interest of researchers. The linear filler is shown to be beneficial in decreasing the charge injection and hindering the formation of charge packs. So, we demonstrate a novel composite with excellent properties. The low-density polyethylene (LDPE) composite with aligned aluminum oxide (Al_2_O_3_) fiber has been prepared in electric field conditions. The direction of the Al_2_O_3_ fiber was parallel to the thickness direction of the LDPE composite. The breakdown strength of the Al_2_O_3_/LDPE composite with 0.2% aligned Al_2_O_3_ fiber was 498 kV/mm, which is higher than other fillers induced. The aligned Al_2_O_3_ fiber has effect on preventing accumulation of space charge and reducing the amount of free electron in the material. In addition, the thermal conductivity of the LDPE composite (0.22 W/m·K) was increased to 0.85 W/m·K when doped with 0.5 wt% aligned Al_2_O_3_ fiber. The present structure provides a new possibility for mass new nanocomposites with excellent microstructures and remarkable functionality.

## 1. Introduction

With the increasing demand for renewable sources of electricity generation, the development of high voltage direct current (HVDC) transmission technologies has been accelerated. However, the operational stability of the power cables needs to be emphasized as the voltage level rises [1,2]. It has been indicated that a high-voltage electric field combined with high temperatures may lead to thermal breakdown and ageing of insulation materials. Hence, enhancing the insulation properties of the polymeric material is one efficient and obvious approach to reduce the failure rate and to further improve the operation stability of power cables [3,4,5]. Many innovative approaches have been explored to improve the electrical properties of polymer dielectrics [6,7,8,9]. On the one hand, multilayered sandwich structures were constructed, and the cross-linking was a treatment for polymers [10,11]. On the other hand, the inorganic particles were induced into polymers [12,13,14]. More specifics would be useful about the other oxide material particles, which in recent years have been studied [15,16,17].

The use of the inorganic particles is shown to be beneficial in decreasing the partial discharges and can mitigate space charge accumulation, which is responsible for improving the electrical properties in these nanocomposites [18,19]. Two reasons can be explained as follows: some researchers considered that the charge transport process has been dependent on shallow trap assisted hops in the direction of the applied field [20,21,22,23]. However, Dissado and Fothergill [24,25] argue that the scattering processes are rather trap distributions that ultimately limit the electron mobility (although the traps may nonetheless serve as scattering centers). So, in this theory, Wang [26] prepared the cellulose composites, and discovered that aligning carbon nanotube fillers parallel to the film surface leads to improved breakdown strength properties. Li [27] demonstrated that the controlled arrangement of pseudo-2D nanofillers and polymer crystals in polyethylene/montmorillonite nanocomposites can be used as an effective approach to achieve nontrivial highly enhanced dielectric performances, far beyond what is feasible with conventional (macroscopic, isotropic) composites. In particular, it is shown that aligned nanofillers can increase the breakdown strength while, at the same time, reducing the leakage current, in these dielectric nanostructured composites. Tomer [28] explored polyethylene/montmorillonite nanocomposites and investigated that the alignment filler can be used to improve the high electric-field breakdown strength and the recoverable energy density. They demonstrated that the addition of high aspect ratio nanofillers arranged parallel to the surface of a dielectric film can provide an optimized distribution of traps and scattering centers that, in turn, can provide the resistance to electric treeing inception and improve the breakdown strength across such a film.

In the context of these ideas, recent investigations indicate that the filler orientation and their spatial distribution play a vital role in determining and improving electrical properties of nanocomposites. Moreover, the aluminum oxide has a tremendous value and also many applications in fusion technology [29,30]. So, it is the focus of this paper to better understand the effect of orientation of high aspect ratio fillers on the electrical properties in LDPE nanocomposites. The LDPE composite material with a certain arrangement of Al_2_O_3_ is prepared by the melt blending method in direct current field conditions. The purpose is to use the directional arrangement of alumina fiber in the LDPE to hinder the movement of carriers and improve the thermal conductivity of the LDPE composite material and reduce the internal material. The accumulation of heat in the material decreased, which could improve the insulating properties of the material.

## 2. Experimental

### 2.1. Materials

The LDPE (LD200BW) with a density of 0.922 g/cm^3^ and melt flow rate of 2.3 g/10 min was purchased from Sinopec Beijing Yanshan Co. (Beijing, China). The spherical nanoalumia (Al_2_O_3_) particles with a diameter of 50–100 nm were modified by 3-aminopropyltriethoxysilane (KH550) and introduced to LDPE, as has been reported in our past work [31], and the composite was named S-Al_2_O_3_/LDPE.

**Figure 1:**
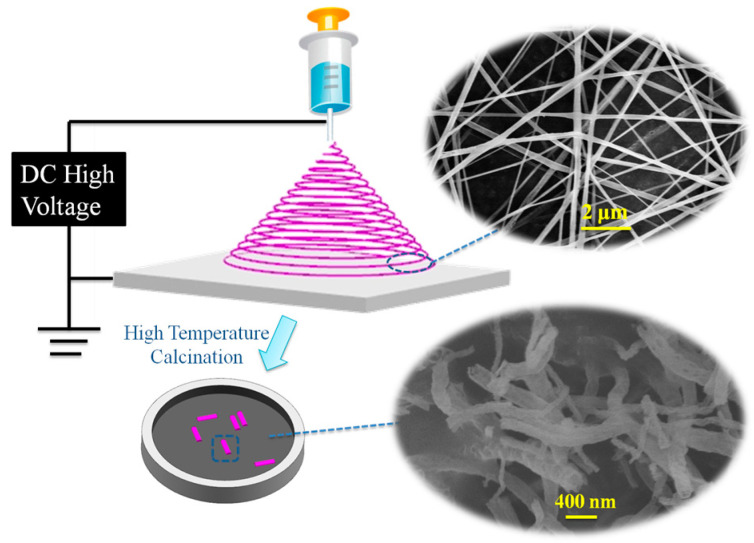
The preparation of Al_2_O_3_ fiber and the micro-structure.

The precursor of Al_2_O_3_ nanofiber was prepared by electrostatic spinning, and the detailed parameters of preparation have been reported in our past work [32]. The Al_2_O_3_ nanofiber were modified by KH-550 before use. Firstly, the Al_2_O_3_ nanofibers were dried in a vacuum oven at 120 °C for 6 h. After cooling to room temperature, 1 g of Al_2_O_3_ nanofibers were added into a 200 mL three necked round-bottomed flask containing 10 mL solvent (ethanol: water = 9:1, vol/vol) and 1 mL KH-550, which was equipped with a mechanical stirrer. A few drops of ammonia were added to adjust the pH of the solution to 9–10. Secondly, the resulting mixture was stirred at room temperature for 30 min, then heated to 60 °C, and kept stirring for 4 h. Thirdly, the mixture was filtered and then quickly washed with deionized water and fresh ethanol. Finally, the product was dried in a vacuum oven at 80 °C for 6 h and then stored in a desiccator. The micro-structure of Al_2_O_3_ nanofiber was investigated by scanning electron microscopy (SEM, S-4700, Hitachi, Japan), and that is as shown in Figure 1.

**Figure 2:**
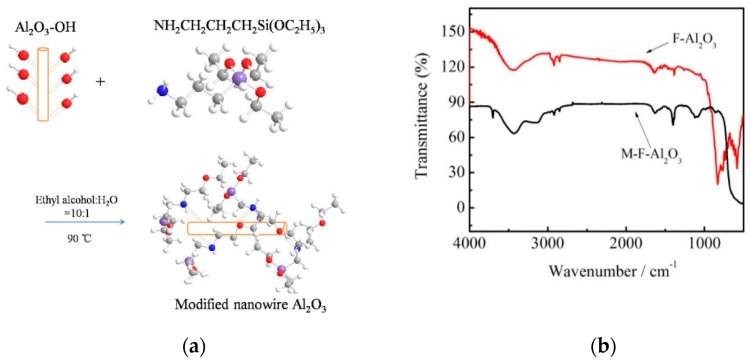
(**a**) Schematic of modified Al_2_O_3_ by KH550, (**b**) FT-IR curves of Al_2_O_3_ fiber and modified Al_2_O_3_ fiber.

Figure 2 a is a schematic diagram of the reaction of Al_2_O_3_ fiber grafting KH550. After the surface is hydroxylated, it is dehydrated and condensed with the amino group of KH550. The amino group of KH550 is combined with the surface of Al_2_O_3_. The repulsive force between the particle and the parent molecular chain.

The modified fiber was analyzed by infrared ray for the grafting condition of the KH-550 modification. As shown in Figure 2b, it can be seen from the figure that the infrared curve of the modified fiber Al_2_O_3_ has an obvious H-N absorption peak at 3600 cm^−1^, which indicates that the -NH_2_ group of KH550 was successfully introduced into the surface of the Al_2_O_3_ fiber.

### 2.2. Preparation of the Nanocomposites

As the research of our team reported [33], the aligned fiber is beneficial to homogenize the electric field. So, the new experiment has been designed with this in mind. The different concentrations of nano-Al_2_O_3_ fiber (0.1 wt%, 0.2 wt%, and 0.5 wt%) were introduced into the LDPE matrix using a HAAKE PolyLab mixer (HAAKE Rheomix 600, Thermo Fisher Scientific, Waltham, MA, Germany). The composite with 0.1 wt% nano-Al_2_O_3_ fiber dispersed randomly is named 0.1%R-Al_2_O_3_/LDPE, and for other concentrations, they are 0.2%R-Al_2_O_3_/LDPE, and 0.5%R-Al_2_O_3_/LDPE, respectively. The R-Al_2_O_3_/LDPE nanocomposites were pressed using panel vulcanizer at 140 °C.

The R-Al_2_O_3_/LDPE thin films were put into the temperature box, which was linked with two electrodes and a DC electrical source. Two polyethylene terephthalate (PET) layers, which are aimed in inhibiting the molten LDPE layer from adhering to the electrodes. The electrons were injected into the molten LDPE composites and accumulated near the Al_2_O_3_ fiber resulting from the polarized effect. It would be explained by two reasons: one is that the Al_2_O_3_ fiber would increase the number of traps. The traps will be bound by electrons. The other reason is that due to the different conductivity of LDPE and Al_2_O_3_ fiber, the electrons are able to transfer near the Al_2_O_3_ fiber. Due to the polarized effect, the polarized charge has tended to accumulate in two ends of Al_2_O_3_ fiber [34,35]. As the accumulation charge increases, the electric field force of the Al_2_O_3_ fiber bearing becomes larger. The direction of Al_2_O_3_ fiber may be changed in this case. The rearranged Al_2_O_3_/LDPE composite has been called aligned-Al_2_O_3_/LDPE (A-Al_2_O_3_/LDPE) composite. The polarizing time of the A-Al_2_O_3_/LDPE composite was 72 h and polarizing voltage was 100 V. When the polarizing process had been accomplished, the temperature of the box would decrease from 140 °C to 25 °C.

Before testing, all the samples were placed in a vacuum oven at 80 °C for 24 h, and then cooled down to room temperature to eliminate their thermal histories. To banish the residual charges, the films were put into two polished copper plates in a vacuum oven at 80 °C for 48 h of short-circuiting.

### 2.3. Characterization

The space charge distribution of the samples was tested by pulsed electro-acoustic measurement (PEA, Shanghai Jiao Tong University, Shanghai, China) under an electric field of 30 kV/mm for 60 min at 25 °C. During the measurement, the samples with a thickness of 350 ± 20 μm were sandwiched between an aluminum electrode with diameter of ~12 cm and one semi-conductive polymer electrode with a diameter of ~2 cm.

The direct current (DC) breakdown strength of the samples was measured using one dielectric strength tester (Ji Lin Province Huayang Equipment Co., Ltd., Jilin, China). The breakdown strength was determined by a progressive electrical stress test. The Weibull distribution of the breakdown strength of the composites was employed to fit the experimental data and determine the characteristic of DC dielectric breakdown strength according to the IEEE Standard 930-2004 [36].

Place a sample with a thickness of 50 μm on a breakdown tester with a boosted rate of 1200 V/s, the diameter of 1 cm of spherical electrode).

The dielectric breakdown strength of the LDPE nanocomposites is analyzed within the framework of Weibull statistics. The Weibull statistical distribution is the most important method for processing data breakdown strength, which reflects the probability of the material at a certain field strength (*E*) or the probability of failure or breakdown at a certain time (*t*). Weibull cumulative distribution breakdown field strength can be described as follows.

(1) $$P=1-exp\left[-{\left(\frac{E}{\alpha }\right)}^{\beta }\right]$$

where *P* is the cumulative probability of the dielectric breakdown *E* is the breakdown strength for testing; *β* is the shape factor related to the dispersion of sample; and *E*_0_ is the characteristic breakdown strength during the cumulative probability of the dielectric breakdown is 63.2%. After twice taking the logarithm, Equation (1) can be deformed as follows.

(2) log[−ln(1−P)]=βlog(E)−βlog(α)

This point log [−ln (1−*P*)] and log (*E*) form a linear relationship in the Cartesian coordinate system. According to IEEE Standard 930-2004, when the number of samples is less than 25, *P* should be computed by the formula as follows.

(3) $$P_{i}=\frac{i-0.44}{n+0.25}\times 100\%$$

where *n* is the breakdown times or voltages in order from smallest to largest and assign them a rank from *i* = 1 to *i* = *n*, *n* is 10.

DC conductivity measurements were conducted in the room temperature range. The DC conduction path testing system, including the high voltage DC power with continuously adjustable output voltage within 0–10 kV, pA ammeter (measuring range of 10^−2^–10^−14^ A), oven temperature (shielding box, the maximum operating temperature) and multi-branching switch, was used to investigate the electrical properties of the insulating materials. The samples with an equal thickness each time were put into the oven. The output voltage was increased progressively. When the current had not changed for 20 min, the data was read every 30 s. The mean data of 40 points were seen as the experiment data.

Morphology of the composites was characterized by scanning electron microscopy (SEM, S-4700, Hitachi, Japan) at an accelerating voltage of 8 kV and 20 kV.

The in-plane thermal diffusivity (*α*) of the composites was performed on a NETZSCH LFA467 laser-flash diffusivity thermal analyzer. The specimens were 25 mm in diameter and around 350 μm in thickness, spray coated with a thin layer of fine graphite powder at both sides. The in-plane thermal conductivity is calculated from the equation.

(4) λ(T)=α(T)ρ(T)c(T) 

where *ρ*(*T*) is the density of the composite and the specific heat capacity *c*(*T*) is the is obtained by DSC.

The samples were measured by differential scanning calorimetry (DSC-60, SHIMADZU Company). The weights of the samples were approximately 4 mg. The experiments were performed in the temperature range from 50 °C to 200 °C under N_2_ atmosphere with the rate of 10 °C/min, and then decreased to 50 °C.

The average specific heat capacity at the target temperature is defined in the Equation (5) [37].

(5) $$Cp=\frac{Q_{sample}-Q_{0}}{m_{sample}\times \Delta T}$$

where *Q_sample_* is the heat exchange into the sample, *Q*_0_ is the heat exchange obtained during the blank experiment, *m_sample_* is the mass of the sample and Δ*T* the temperature interval (in this study Δ*T* is equal to 10 °C). The experiment data of samples have been listed in the Table 1. 

## 3. Results

### 3.1. Microstructures

**Figure 3:**
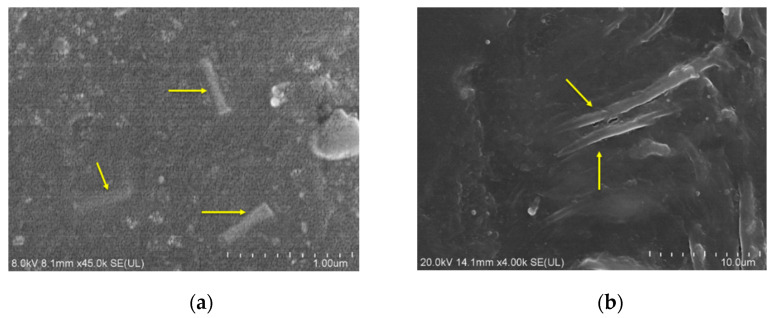
SEM micrographs of Al_2_O_3_/LDPE composites (**a**) randomly dispersed, (**b**) array dispersed.

The microstructures of R-Al_2_O_3_/LDPE and A-Al_2_O_3_/LDPE composites were observed by SEM. The samples were quenched in liquid nitrogen before the observation. The cross-section SEM images of R-Al_2_O_3_/LDPE and A-Al_2_O_3_/LDPE composites are shown in Figure 3. As shown in Figure 3a, Al_2_O_3_ fibers are randomly dispersing in LDPE. Figure 3b reveals that Al_2_O_3_ fibers are arranging almost parallel to the surface of LDPE composites. It was proven that the rearrangement of Al_2_O_3_ fibers could form a neat array structure under the electric field for a long time.

### 3.2. Dielectric Breakdown Strength

**Figure 4:**
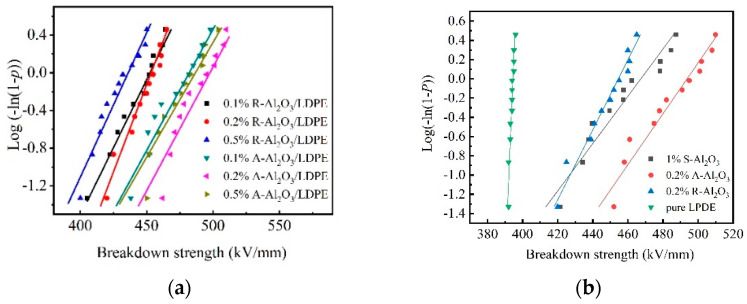
The breakdown strength of Al_2_O_3_/LDPE composites with the thickness of 50 ± 5 μm (**a**) different content of R-Al_2_O_3_/LDPE and A-Al_2_O_3_/LDPE; (**b**) pure LPDE and three different types of Al_2_O_3_/LDPE composites.

The dielectric breakdown strength of the LDPE nanocomposites is analyzed within the framework of Weibull statistics. Weibull statistical distribution is one important method to analyze the breakdown strength of materials. The thickness of the composite is 50 ± 5 μm. Figure 4 is the breakdown strength of the A-Al_2_O_3_/LDPE composite and R-Al_2_O_3_/LDPE composite with different contents. As shown in the Figure 4a, the breakdown strength of 0.2% A-Al_2_O_3_/LDPE composites is 498 kV/mm, while that of 0.1% A-Al_2_O_3_/LDPE composites and the 0.5% A-Al_2_O_3_/LDPE composite are 480 kV/mm and 489 kV/mm, respectively. The breakdown strength of the R-Al_2_O_3_/LDPE composite is relatively lower. The breakdown strength of 0.1% R-Al_2_O_3_/LDPE is 447 kV/mm, and that of 0.2% R-Al_2_O_3_/LDPE is 449 kV/mm, and that of 0.5% R-Al_2_O_3_/LDPE is only 428 kV/mm. Figure 4b shows the breakdown strength of Al_2_O_3_/LDPE composites with the different types of Al_2_O_3_ filling. As compared the breakdown strength of the samples, the breakdown strength of pure LDPE is only 392 kV/mm.

The sample with orient-Al_2_O_3_ fiber exhibits the highest breakdown strength. The oriented filler should frustrate the formation of electrical treeing. The reason may be that the Al_2_O_3_ fiber in the A-Al_2_O_3_/LDPE composite is oriented, and the arrangement direction is parallel to the surface of LDPE composites. When the charge is injected from the electrode to the inside of the material, the transport path of the charges will be blocked by these directional alignment fibers. The charges would be trapped by the traps near the fiber, and some charges would change their transport path. So, these directional alignment fibers would increase the migration path of the charges in the material and reduce the formation of effective conductive pathways. There is a possibility that the conductivity of the interface between the Al_2_O_3_ fiber and the pure LDPE is higher than that of the LDPE, and the carrier is more likely to transport near the Al_2_O_3_ fiber. The fibers are arranged in parallel and perpendicular to the transport path. Reducing the formation of charge packets in the material is important to improve the breakdown strength of the material.

### 3.3. DC Conductivity

**Figure 5:**
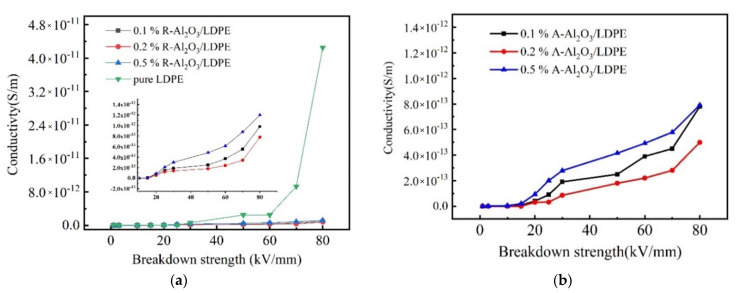
DC conductivity of (**a**) R−Al_2_O_3_/LDPE and pure LDPE, (**b**) A-Al_2_O_3_/LDPE.

In order to research the free electron in the material, the DC conductivity has been measured. The conductivities of the random-dispersed nanocomposites are shown in Figure 5a. It can be seen that the conductivities of pure LDPE are generally larger than that of the R-Al_2_O_3_/LDPE composite at the same electrical strength. The conductivities of the R-Al_2_O_3_/LDPE composite at 20–80 kV/mm are described in Figure 5a. It is worth noting that the conductivity of the R-Al_2_O_3_/LDPE composite is greatly reduced and its value approaches 4 × 10^−12^ S/m, thus implying that the Al_2_O_3_ fiber filled in nanocomposite can effectively inhibit the current increase.

Compared the DC conductivity of AAl_2_O_3_/LDPE composites, R-Al_2_O_3_/LDPE composite, and pure LDPE, the A-Al_2_O_3_/LDPE (0.1%, 0.2%, 0.5%) is significantly lower than that of R-Al_2_O_3_/LDPE with the corresponding contents in the range from 1 kV/mm to 80 kV/mm. Figure 5b was shown that the conductivity of 0.2% A-Al_2_O_3_/LDPE was 4.0 × 10^−13^ S·m^−1^ under the electric field of 80 kV/mm, and as shown in Figure 5a, the conductivity of 0.2% R-Al_2_O_3_/LDPE is 8.0 × 10^−13^ S·m^−1^. When the electric filed strength is 50 kV/mm, the increased rate of conductivity of R-Al_2_O_3_/LDPE is faster than that of A-Al_2_O_3_/LDPE. Therefore, it was confirmed that the transport velocity of carriers in the A-Al_2_O_3_/LDPE composites was decreased, which would result from the fact that the array arrangement of the Al_2_O_3_ fiber is more beneficial to block the migration of the carriers and reduce the rate of carrier transport in the material.

**Figure 6:**
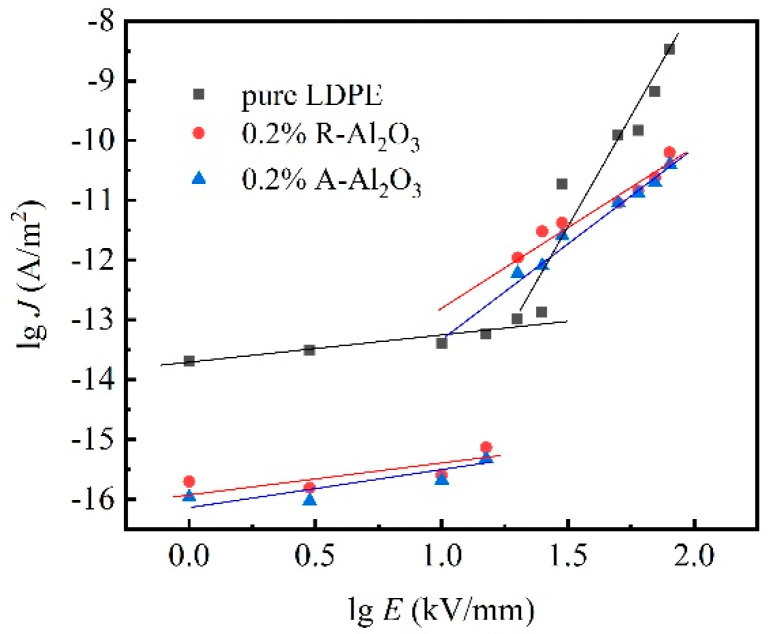
Current densities of pure LDPE, R-Al_2_O_3_/LDPE and A-Al_2_O_3_/LDPE depended on electrical strength.

Figure 6 shows the electrical strength dependency on current densities of 0.2% R-Al_2_O_3_, 0.2% A-Al_2_O_3_ and pure LDPE composites. As shown in Figure 6, the electric field thresholds which linked the Ohmic region and non-Ohmic region are decreasing as Al_2_O_3_ fiber filled. Based on the Space Limited Current (SCLC) theory [38,39], the injecting charge has nearly been trapped in the Ohmic region. The injecting charge would not contribute to the current increasing. For the non-Ohmic region, the trapping charge in the composite will be detrapping, and the injecting charges will increase the current density. As the electric field increased, the injecting charge amount in the pure LDPE increases and the electron transport is more active.

### 3.4. Space Charge

In order to investigate the accumulation and dissipation of charge in the Al_2_O_3_/LDPE composites in detail, the sample with thickness of 350 ± 20 μm had been measured by PEA method.

**Figure 7:**
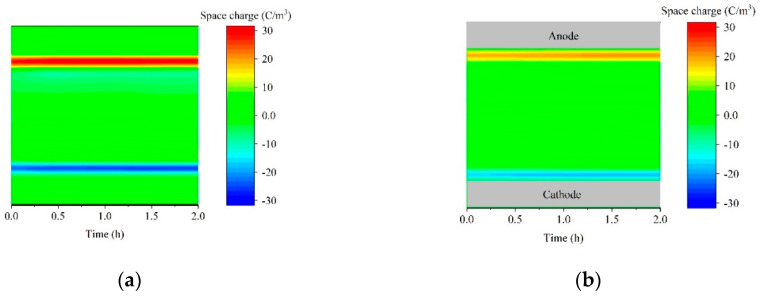

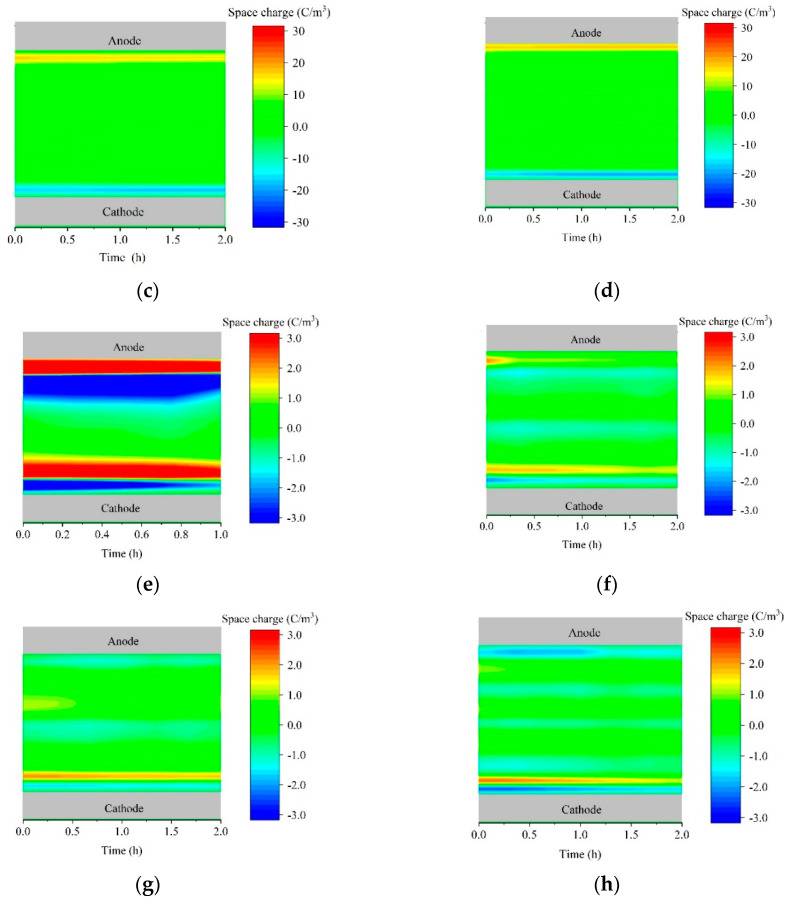
Effect of A-Al_2_O_3_/LDPE on dynamics space charge injection and suppression (**a**) pure LDPE, (**b**) 0.1% A-Al_2_O_3_/LDPE, (**c**) 0.2% A-Al_2_O_3_/LDPE, (**d**) 0.5% A-Al_2_O_3_/LDPE, Dependences of the relaxation time in the decay of total trapped charge in (**e**) pure LDPE, (**f**) 0.1% A-Al_2_O_3_/LDPE, (**g**) 0.2% A-Al_2_O_3_/LDPE, (**h**) 0.5% A-Al_2_O_3_/LDPE under 30 kV/mm upon the electrical field before short circuiting.

Figure 7 shows the injecting and decaying effect of the dynamics space charge of A-Al_2_O_3_/LDPE. Figure 7a–d is the space charge profiles of A-Al_2_O_3_/LDPE composites (0, 0.1%, 0.2%, and 0.5%) under 30 kV/mm stressing for 2 h, respectively. It can be seen that the charge accumulated in the electrode. The accumulation of space charge has been increased as time passes. The amount of charge in the A-Al_2_O_3_/LDPE composite is smaller than that of pure LDPE. This indicates that the array arrangement can effectively inhibit the formation of heterogeneous charges, resulting in a reduction in the number of charges inside the material. Especially, the A-Al_2_O_3_/LDPE composite with 0.2% A-Al_2_O_3_ loading, which has just a little charge.

Figure 7e–h have shown the charge dissipation processes of composites in short-circuit after pressing for 2 h under a 30 kV/mm electric field. It revealed that the charge dissipation rate of A-Al_2_O_3_/LDPE composites is significantly faster than that of pure LDPE. For pure LDPE, the accumulation charge in the material, some charge overflows from the electrode, and some charges diffuse into the material. So much charge remained in the material, which would be a problem for practical application. However, the charge in the 0.2% A-Al_2_O_3_/LDPE composites dissipated very fast. After decaying for 1 h, there is no charge in the material. The orientation structure succeeds in changing the transport mode of carriers in the medium, reducing the trap level and making the carriers easy to transport along the direction parallel to the thickness, effectively suppressing the injection of carriers along the thickness direction [40].

### 3.5. Thermal Conductivity

**Figure 8:**
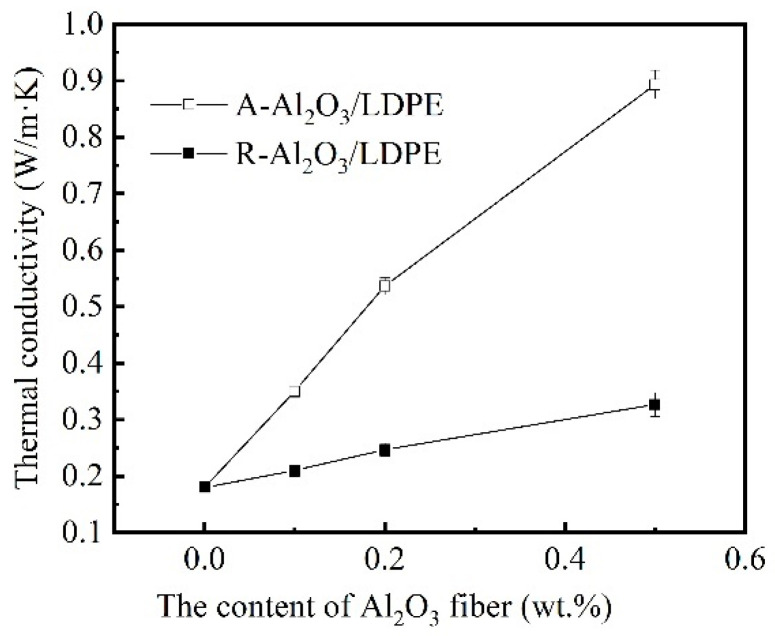
The thermal conductivity of the nanocomposites containing various amounts of Al_2_O_3_ fiber.

Figure 8 shows the thermal conductivity of the composites containing various amounts of Al_2_O_3_ fiber. It can be seen that the thermal conductivity of composites increased significantly with the content of Al_2_O_3_ fiber increasing. The thermal conductivity of LDPE with 0.5 wt% A-Al_2_O_3_ loading is 0.85 W/m·K, which is 3 times higher than that of pure LDPE (0.22 W/m·K). The thermal conductivities of the R-Al_2_O_3_/LDPE composite are lower than that of the A-Al_2_O_3_/LDPE composite.

**Figure 9:**
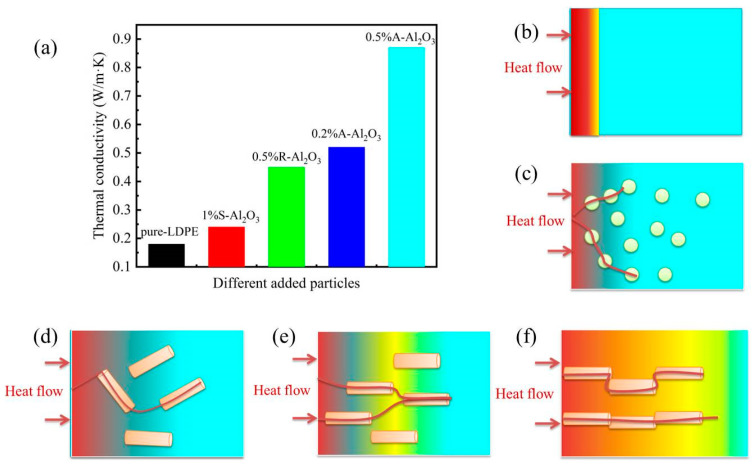
The thermal conductivity of Al_2_O_3_/LDPE with different particles and the heat flow (**a**) the diagram of heat flow (**b**) pure LDPE, (**c**) S-Al_2_O_3_/LDPE (**d**) R-Al_2_O_3_/LDPE (**e**) 0.1%A-Al2O3/LDPE (**f**) 0.1%A-Al2O3/LDPE composite.

The thermal conductivity of Al_2_O_3_/LDPE with different particles and the diagram ofheat flow are shown in Figure 9. This may be explained by the reasons shown in Figure 9b–f. (i) The LDPE matrix is poor conductor of heat; the forming of a heat path is beneficial to the heat conductivity. (ii) The spheroidal particles with a small amount have lower thermal conductivity. (iii) Along with the increase of the content of A-Al_2_O_3_ fiber, the local concentration of the Al_2_O_3_ fiber forming the co-continuous structure is a better conductor of heat.

The directional arrangement of alumina fiber in the LDPE hindered the movement of carriers. The Al_2_O_3_ fiber of A-Al_2_O_3_/LDPE composite could increase the thermal conductivity of the LDPE composite material and reduce the internal material accumulation. The dissipation of heat in the material could decrease the rate of carrier transporting, which is beneficial in improving the insulating properties of the material.

## 4. Conclusions

In summary, the surface grafting of Al_2_O_3_ fibers by KH550 successfully increased the compatibility of the Al_2_O_3_ fiber with LDPE. In the electric field conditions, the Al_2_O_3_ fiber in the molten Al_2_O_3_/LDPE composite has been rearranged. The breakdown strength of Al_2_O_3_ on LDPE composites with different morphologies has shown that the array-structured Al_2_O_3_ has improved the breakdown strength of LDPE from 470 kV/mm to 498 kV/mm. The DC conductivity of 0.2% A-Al_2_O_3_/LDPE composites had been slow with the electric field increase, and for which conductivity was 4 × 10^−13^ S/m. The reason may be that the parallel Al_2_O_3_ fiber in the composites could reduce the accumulation of space charge.

## Figures and Tables

**Table 1:** Experiment data of samples.

Samlpe	*m* (kg)	Δ*Q* (J)	*C* (J/kg·K)	*ρ* (g/cm^3^)	*α* (mm^2^/s)
0.1A	4.07 × 10^−6^	3.71 × 10^−3^	903.9	1.21	0.32
0.2A	4.11 × 10^−6^	3.65 × 10^−3^	890.4	1.24	0.48
0.5A	4.15 × 10^−6^	3.35 × 10^−3^	818.3	1.25	0.87
LDPE	4.01 × 10^−6^	4.39 × 10^−3^	1071.4	1.12	0.15
0.1R	4.14 × 10^−6^	3.59 × 10^−3^	875.1	1.2	0.2
0.2R	4.01 × 10^−6^	3.67 × 10^−3^	894.1	1.22	0.22
0.5R	4.06 × 10^−6^	3.59 × 10^−3^	875.8	1.26	0.29

## Data Availability

The data presented in this study are available on request from the corresponding author.
